# The efficacy of bevacizumab combined with platinum-containing chemotherapy in the treatment of advanced non-small cell lung cancer in China: a systematic review and meta-analysis of randomized clinical trials

**DOI:** 10.3389/fphar.2024.1293039

**Published:** 2024-01-22

**Authors:** Guangsu Han, Chenlu Li, Ping Yi

**Affiliations:** ^1^ Institute of Integrated Traditional Chinese and Western Medicine, Tongji Hospital, Tongji Medical College, Huazhong University of Science and Technology, Wuhan, China; ^2^ Department of Integrated Traditional Chinese and Western Medicine, Tongji Hospital, Tongji Medical College, Huazhong University of Science and Technology, Wuhan, China

**Keywords:** bevacizumab, non-small cell lung cancer, chemotherapy, meta-analysis, systematic review

## Abstract

**Background:** Platinum-based dual-drug first-line chemotherapy is commonly employed in the treatment of patients with advanced non-small cell lung cancer (NSCLC), although its clinical efficacy is limited. Bevacizumab can antagonize vascular endothelial cell growth factor (VEGF), which inhibit tumor angiogenesis and prevent tumor invasion and development. However, a comprehensive meta-analysis evaluating the effectiveness and safety of combining bevacizumab with platinum-based chemotherapy in advanced NSCLC patients is lacking.

**Methods:** Randomized controlled trials (RCTs) investigating the combination therapy of bevacizumab and platinum-based chemotherapy for treating advanced NSCLC were searched across six databases. Data on objective response rate (ORR), disease control rate (DCR), 1-year survival rate, 2-year survival rate, 3-year survival rate, VEGF levels, and side effects were synthesized. Relative risk degree (RR) along with 95% confidence interval (CI) was used as statistical analysis measures for binary outcomes while continuous variables were analyzed using mean difference (MD) along with 95% CI. Heterogeneity was evaluated by Chi-squared and I^2^ tests. If there was heterogeneity, subgroup analysis was performed. Sensitivity analysis of the main outcome measures and assessment of publication bias were also performed.

**Results:** According to our screening criteria, a total of Forty-nine RCTs were included, involving data from 4268 patients. The results of this analysis showed that compared with platinum-containing chemotherapy alone, bevacizumab combined with platinum-containing chemotherapy significantly improved ORR (RR [95% CI], 1.53 [1.44, 1.63], *p* < 0.00001), DCR (RR [95% CI], 1.24 [1.19, 1.29], *p* < 0.0001), 1-year survival rate (RR [95% CI], 1.34 [1.15, 1.57], *p* = 0.0003), 2-year survival rate (RR [95% CI], 2.16 [1.35, 3.43], *p* = 0.001), 3-year survival rate (RR [95% CI], 2.00 [1.21, 3.30], *p* = 0.007). In addition, bevacizumab with platinum-containing chemotherapy observably decreased the VEGF levels (RR [95% CI], −67.35 [−91.46, −43.25], *p* < 0.00001).

**Conclusion:** Combination therapy involving bevacizumab demonstrated improved antitumor effects compared to chemotherapy alone in terms of ORR, DCR, 1-year survival rate, 2-year survival rate, 3-year survival rate, and VEGF levels without an increased incidence of adverse reactions. These analyses’ results can provide clinicians guidance when selecting appropriate treatments for patients diagnosed with advanced non-small cell lung cancer.

## Introduction

Lung cancer is the most common malignancy worldwide and has the highest mortality rate of all cancers. It is estimated that by 2030, it will cause 2 million deaths a year worldwide (Mattiuzzi and Lippi, 2019). Lung cancer can be classified into small-cell lung cancer (SCLC) and non-small cell lung cancer (NSCLC). NSCLC, as a group of histological subtypes, accounts for 80 percent of total lung cancer diagnoses (Thai et al., 2021). In the early stage of non-small cell lung cancer, there is a lack, leading to most diagnosed at an advanced stage and missing the opportunity for radical surgery. In the past 2 decades, the emergence of targeted therapy and the application of immunotherapy have made substantial progress in the treatment of NSCLC. Antibodies to CTLA-4, PD-1, PD-L1 and other immune checkpoints have been approved by the US FDA for the treatment of a variety of tumors, including non-small cell lung cancer (Le et al., 2017). It has been included in the latest global cancer treatment guidelines (Planchard et al., 2018). Nevertheless, the existence of drug resistance still makes its accessibility and effectiveness limited (Herbst et al., 2018). Platinum-based dual-drug first-line chemotherapy is commonly used for advanced NSCLC treatment; however, patients exhibit low sensitivity to this approach with an overall effective rate ranging from 25.0% to 35.0% (Chino et al., 2016). Therefore, it has become imperative to identify an efficient comprehensive treatment method for advanced NSCLC from traditional chemotherapy, targeted and immunotherapy have emerged as novel treatment modalities.

The formation of blood vessels plays an important role in the growth and metastasis of tumors, and vascular endothelial growth factor (VEGF) is named for its ability to promote endothelial cell proliferation and lumen formation, and its overexpression is associated with poor prognosis in patients with non-small cell lung cancer (Niki et al., 2017). Bevacizumab, a monoclonal antibody with high affinity towards VEGF, is frequently employed in the management of ovarian cancer, advanced metastatic colorectal cancer, and other diseases (Fan et al., 2019). By competitively binding with by tumor tissues, bevacizumab inhibits angiogenesis within these tissues while preventing tumor cell proliferation. Additionally, bevacizumab improves blood vessel function, microenvironment and enhances drug concentration within cancer tissues thereby achieving favorable therapeutic effects (Zheng et al., 2018). Clinical studies have demonstrated that bevacizumab confers significant survival benefits in patients with advanced NSCLC (Yang et al., 2018). There have been similar meta-analyses of bevacizumab combined with chemotherapy in the treatment of non-small cell lung cancer, but some of them only involved overall survival (OS), progression-free survival (PFS) and side effects (Botrel et al., 2011), and some lacked further verification regarding disease control rate (DCR) which serves as an important indicator (Zhou et al., 2021). The objective of this systematic review and meta-analysis was to comprehensively evaluate the efficacy and safety of bevacizumab and platinum-containing chemotherapy in the treatment of advanced NSCLC by including more recent studies.

## Materials and methods

### Search strategy

We followed the PRISMA (Preferred Reporting Items for Systematic reviews and Meta-Analyses) checklist for systematic reviews and meta-analyses (Liberati et al., 2009). We conducted a systematic search for randomized controlled trials investigating the combination of bevacizumab and platinum-containing chemotherapy in patients with advanced NSCLC published in six databases from January 2018 to April 2023, including PubMed, Web of Science, the Cochrane Library, Wan Fang Database, Chinese VIP Information (VIP) and China National Knowledge Infrastructure (CNKI).

The strategy of combining subject words and free words is adopted to search. In the Chinese database, the following words are used in a combination manner: “Bei fazhu,” “feixiao xibao feiai” and “hualiao”; for the English databases, the text terms we use include “Bevacizumab,” “Carcinoma, Non-Small-Cell Lung” and “Drug Therapy”

### Selection criteria

The inclusion criteria were as follows: 1) study design: randomized randomized controlled trials (RCTs). 2) population: pathologically, cytologically or histologically confirmed to have advanced NSCLC. 3) intervention: the control group was treated with platinum-containing chemotherapy and correspondingly, the experimental group was treated with bevacizumab on the basis of the control group. 4) outcome: objective response rate, disease control rate, year survival rate, progression-free survival, overall survival, VEGF levels and treatment-related aside effects.

The exclusion criteria were as follows: 1) non-RCTs including case reports, reviews, animal or cell studies and studies without a control group. 2) The interventions were not bevacizumab and platinum-containing chemotherapy. 3) ambiguous results of statistical methods and research indicators, difficult to extract outcome indicators data, and multiple publications. 4) The patient had small cell lung cancer or early-stage NSCLC.

### Data extraction

Data extraction was carried out independently by two reviewers (Guang Su Han and Chen Lu Li) and differences on study eligibility were resolved by consensus. The key information extracted by the evaluators is as follows: the author, year of publication, the number of participants, intervention details, the TNM stage of NSCLC and relevant outcomes.

### Methodological quality

Two researchers (Guang Su Han and Chen Lu Li) independently evaluated the quality of the included literature using the Cochrane risk of bias tool. The following six criteria were evaluated: 1) random sequence generation and allocation concealment; 2) blinding of participants and personnel; 3) blinding of outcome assessment; 4) incomplete outcome data; 5) selective reporting; 6) other bias. Differences arising in the process of quality evaluation were resolved through mutual consultation and discussion.

### Data synthesis and analysis

In this study, Review Manager (ver. 5.3) and STATA (ver.14) software were used for statistical analysis. RR and 95%CI were used as effect analysis statistics to treat binary outcomes. Continuous variables were analyzed by MD and 95%CI. *p* < 0.05 means the difference is statistically significant. Heterogeneity was evaluated by Chi-squared and I^2^ tests. If *p* > 0.1 and I^2^ < 50%, indicating that there was no statistical heterogeneity among the studies, and a fixed effect model was used for meta-analysis; If the heterogeneity was moderate or severe (*p* ≤ 0.1 or I^2^ ≥ 50%), the factors that might lead to heterogeneity were analyzed by subgroup analysis. If there was heterogeneity between the two studies and the clinical difference was not statistically significant, we choose a random-effect model. Then we performed sensitivity analyses to account for the effect of changing the study mode on the results of the pooled analysis. Since more than 10 studies were included, publication bias was identified using Begg’s tests and *p*-value > 0.05 was considered as no publication bias.

## Results

### Study inclusions

**FIGURE 1:**
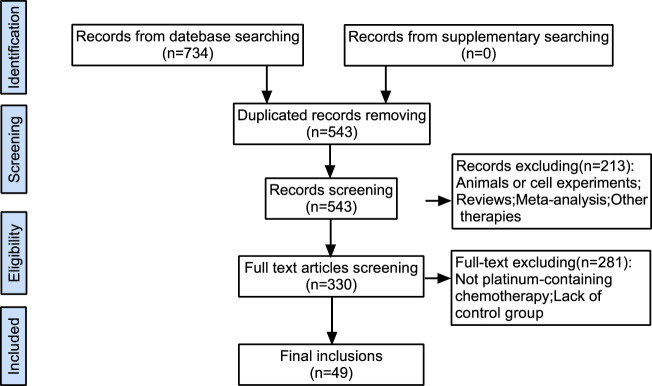
Flow chart of study selection process.

734 literatures that meet the screening requirements were initially searched from the database, including 191 repeated records (Figure 1). After that, 330 studies were included by reviewing the titles and abstracts of the remaining 543 studies and removing 213 reviews, animal or cell experiments, case reports, and other inconsistent literature. Finally, studies with no control group or platinum-containing chemotherapy regimen were excluded (*n* = 281), and 49 RCTs were selected in this systematic analysis (Dang et al., 2018; Ding and Zhu, 2018; Duan et al., 2018; Luo and Liu, 2018; Gui, 2019; Ma, 2019; Chen, 2020; Duan, 2020; Han, 2020; Li, 2020; Liao, 2020; Chang et al., 2021; Dai et al., 2021; Du, 2021; Fan, 2021; Huang and Du, 2021; Mao, 2021; Pan and Xia, 2021; Chen et al., 2022a; Liu et al., 2022a; Chen et al., 2022b; Liu et al., 2022b; He, 2022; Li et al., 2022; Liu, 2022; Ning et al., 2022; Shi, 2022; Song et al., 2019; Sun et al., 2019; Xu, 2019; Zhai et al., 2019; Wang et al., 2020a; Wang et al., 2020b; Song et al., 2020; Wang and Qian, 2020; Wu, 2020; Zhang, 2020; Zhang and Wang, 2020; Song, 2021; Wang, 2021; Wang and Zheng, 2021; Yuan, 2021; Tian and Wang, 2022; Wu et al., 2022; Yang, 2022; Ye et al., 2022; Zeng and Li, 2022; Sun, 2023; Zhou and Rong, 2023).

### Characteristics of the studies

A total of forty-nine studies were included in the evaluation, and 4268 patients were studied. All RCTs originated from China. The year of publication was between 2018 and 2023. The characteristics of patients in the selected studies include age, gender, clinical stage, chemotherapy regimen, and treatment indicators, which are summarized in Table 1.

**TABLE 1:** Characteristics of RCTs included in the study. (GP, Gemcitabine; AP, Pemetrexed; TP/DDP/PP, Taxol; DP, Docetaxel; ①Efficacy (RECIST); ②Efficacy (WHO); ③Efficacy (Not mentioned); ④1-year survival; ⑤2-year survival; ⑥3-year survival; ⑦Progression-free survival; ⑧Overall survival; ⑨VEGF; ⑩Adverse reaction).

Study	Sample size (E/C)	Sex (male/female) (C E)	Age	Clinical stage	Control group (C)	Experiment group (E)	First treatment	Outcome
Chang et al., 2021	60/60	34/26 32/28	50.22 ± 2.22 50.02 ± 2.19	Ⅲb,Ⅳ	TP	Bevacizumab + TP	NO	①⑨⑩
Chen 2020	37/37	23/14 24/13	55.10 ± 6.82 54.86 ± 7.13	Ⅲb,Ⅳ	GP	Bevacizumab + GP	NO	③⑨⑩
Chen et al., 2022	50/50	34/16 32/18	71.51 ± 7.68 72.46 ± 7.52	Ⅲ,Ⅳ	AP	Bevacizumab + AP	YES	①⑩
Chen hailong et al., 2022	43/43	25/18 23/20	60.25 ± 4.06 59.76 ± 4.11	Ⅲ,Ⅳ	AP	Bevacizumab + AP	YES	①⑨⑩
Dai et al., 2021	40/40	23/17 25/15	58.69 ± 4.36 58.57 ± 5.42	Ⅲb,Ⅳ	TP	Bevacizumab + TP	YES	①⑨⑩
Dang et al., 2018	31/31	17/14 18/13	55.17 ± 3.08 54.82 ± 3.49	Ⅲb,Ⅳ	AP	Bevacizumab + AP	NO	①⑦⑨⑩
Ding and Zhu 2018	45/45	26/19 27/18	55.53 ± 6.26 55.42 ± 6.14	Ⅲb,Ⅳ	TP	Bevacizumab + TP	NO	②⑦⑧⑩
Du 2021	72/72	46/26 45/27	52.36 ± 1.38 52.15 ± 1.34	advanced	AP	Bevacizumab + AP	NO	①
Duan 2020	44/44	26/18 27/17	61.8 ± 5.7 62.1 ± 5.8	advanced	TP	Bevacizumab + TP	YES	①⑩
Duan et al., 2018	93/93	52/41 55/38	58.3 ± 10.6 57.9 ± 11.2	Ⅲb,Ⅳ	GP	Bevacizumab + GP	YES	①⑨⑩
Fan 2021	19/19	12/7 13/6	60.55 ± 6.74 61.52 ± 6.35	advanced	TP	Bevacizumab + TP	NO	②⑩
Gui 2019	42/42	28/14 25/17	59.8 ± 5.1 60.8 ± 5.5	Ⅲa,Ⅲb	TP	Bevacizumab + TP	YES	①⑦⑧⑩
Han 2020	26/26	13/13 12/14	45.52 ± 4.33 46.21 ± 3.26	Ⅲ,Ⅳ	AP	Bevacizumab + AP	YES	②
He 2022	45/45	25/20 27/18	50.98 ± 3.34 51.06 ± 3.45	Ⅲ,Ⅳ	TP	Bevacizumab + TP	YES	③⑩
Huang and Du 2021	33/32	21/11 21/12	45.18 ± 5.34 45.21 ± 5.06	advanced	AP/DP	Bevacizumab + AP/DP	YES	③⑩
Li 2020	50/50	23/27 22/28	59.6 ± 10.3 59.6 ± 10.3	Ⅲ,Ⅳ	TP	Bevacizumab + TP	NO	①
Li et al., 2022	40/40	19/21 22/18	46.97 ± 6.71 47.13 ± 6.54	Ⅲ,Ⅳ	GP	Bevacizumab + GP	YES	①⑨⑩
Liao 2020	40/40	30/10 28/12	58.69 ± 5.23 58.63 ± 5.24	advanced	TP	Bevacizumab + TP	NO	③
Liu 2022	36/36	21/15 20/16	51/59 ± 9.63 51.56 ± 9.69	Ⅲ,Ⅳ	AP	Bevacizumab + AP	NO	①⑨⑩
Liu et al., 2022	36/36	20/16 19/17	54.89 ± 6.56 54.96 ± 6.68	Ⅲb,Ⅳ	GP	Bevacizumab + GP	NO	③⑩
Liu zhen et al., 2022	38/42	27/15 25/13	63.47 ± 5.15 63.42 ± 5.13	Ⅲ,Ⅳ	TP	Bevacizumab + TP	NO	①⑨⑩
Luo and Liu 2018	51/51	33/18 32/19	50.29 ± 8.53 50.83 ± 8.61	Ⅲb,Ⅳ	AP	Bevacizumab + AP	YES	①⑦⑩
Ma 2019	60/60	37/23 34/26	64.9 ± 4.7 64.2 ± 4.2	Ⅲ,Ⅳ	TP	Bevacizumab + TP	YES	①⑩
Mao 2021	25/25	13/12 14/11	67.19 ± 3.27 67.43 ± 3.52	advanced	AP	Bevacizumab + AP	NO	①⑩
Ning et al., 2022	75/75	40/35 43/32	58.33 ± 6.45 58.90 ± 7.08	Ⅲ,Ⅳ	TP	Bevacizumab + TP	YES	②⑨⑩
Pan and Xia 2021	40/40	26/14 28/12	64.85 ± 9.30 65.74 ± 8.28	Ⅲ,Ⅳ	AP	Bevacizumab + AP	NO	①⑩
Shi 2022	41/41	23/18 22/19	67.67 ± 4.44 67.20 ± 4.59	Ⅲb,Ⅳ	TP	Bevacizumab + TP	NO	①⑩
Song 2021	31/31	17/14 16/15	68.14 ± 3.54 67.54 ± 3.19	advanced	AP	Bevacizumab + AP	NO	①④⑥⑧⑩
Song et al., 2019	30/30	17/13 16/14	54.18 ± 8.42 54.36 ± 8.38	Ⅲ,Ⅳ	AP	Bevacizumab + AP	NO	①④⑤⑥⑨⑩
Song et al., 2020	41/39	28/11 26/15	53.72 ± 6.48 55.45 ± 7.12	Ⅲ,Ⅳ	TP	Bevacizumab + TP	NO	②⑨⑩
Sun 2023	41/41	23/18 22/19	66.22 ± 6.82 65.78 ± 7.28	Ⅲ,Ⅳ	PP	Bevacizumab + PP	NO	①⑩
Sun et al., 2019	60/60	41/19 39/21	60.9 ± 6.8 61.1 ± 6.7	Ⅲ,Ⅳ	TP	Bevacizumab + TP	NO	①⑨⑩
Tian and Wang 2022	51/51	31/20 32/19	60.17 ± 10.45 60.29 ± 10.75	Ⅲb,Ⅳ	TP	Bevacizumab + TP	YES	①⑨⑩
Wang 2021	43/43	23/20 25/18	47.79 ± 2.63 47.82 ± 2.59	advanced	AP	Bevacizumab + AP	NO	①⑩
Wang and Qian 2020	30/30	18/12 19/11	61.25 ± 4.87 61.26 ± 4.84	Ⅲb,Ⅳ	DP	Bevacizumab + DP	NO	①⑩
Wang and Zheng 2021	28/28	17/11 18/10	57.08 ± 8.48 56.47 ± 8.55	Ⅲb,Ⅳ	AP/DP/GP	Bevacizumab + AP/DP/GP	NO	③⑩
Wang daofeng et al., 2020	32/32	20/12 21/11	57.96 ± 7.94 58.45 ± 7.71	Ⅲb,Ⅳ	DDP	Bevacizumab + DDP	NO	③⑩
Wang et al., 2020	36/36	25/11 22/14	69.13 ± 7.89 68.59 ± 8.65	Ⅲb,Ⅳ	DDP	Bevacizumab + DDP	NO	③④⑤⑨⑩
Wu 2020	30/30	12/18 14/16	42.68 ± 2.62 43.34 ± 2.24	Ⅲb,Ⅳ	TP	Bevacizumab + TP	NO	③⑩
Wu et al., 2022	50/50	28/22 29/21	61.2 ± 4.1 61.8 ± 3.4	advanced	AP	Bevacizumab + AP	YES	①⑦⑩
Xu 2019	33/33	18/15 17/16	62.34 ± 5.64 61.28 ± 5.34	advanced	AP	Bevacizumab + AP	YES	③⑩
Yang 2022	73/73	34/39 36/37	63.20 ± 6.55 64.10 ± 6.70	Ⅲ,Ⅳ	AP/TP	Bevacizumab + AP/TP	NO	③⑨⑩
Ye et al., 2022	50/50	35/15 37/11	55.62 ± 5.37 54.39 ± 6.21	advanced	TP	Bevacizumab + TP	YES	①⑦⑨⑩
Yuan 2021	101/101	72/29 75/26	55.38 ± 2.61 56.01 ± 4.42	Ⅲ,Ⅳ	TP	Bevacizumab + TP	NO	①⑩
Zeng and Li 2022	25/25	14/11 13/12	56.45 ± 13.29 45.94 ± 13.41	Ⅲ,Ⅳ	TP	Bevacizumab + TP	YES	①⑨⑩
Zhai et al., 2019	44/44	27/17 29/15	71.03 ± 8.11 70.14 ± 9.25	Ⅲb,Ⅳ	TP	Bevacizumab + TP	NO	①④⑤⑩
Zhang 2020	30/30	17/13 18/12	54.73 ± 5.10 54.76 ± 5.15	Ⅲ,Ⅳ	AP	Bevacizumab + AP	NO	①
Zhang and Wang 2020	30/30	17/13 16/14	46.12 ± 8.01 46.23 ± 7.86	Ⅲb,Ⅳ	AP	Bevacizumab + AP	NO	①⑦⑨
Zhou and Rong 2023	33/32	22/10 23/10	55.31 ± 3.23 55.52 ± 3.17	Ⅲ,Ⅳ	TP	Bevacizumab + TP	NO	①⑦⑧⑨⑩

### Quality assessment

**FIGURE 2:**
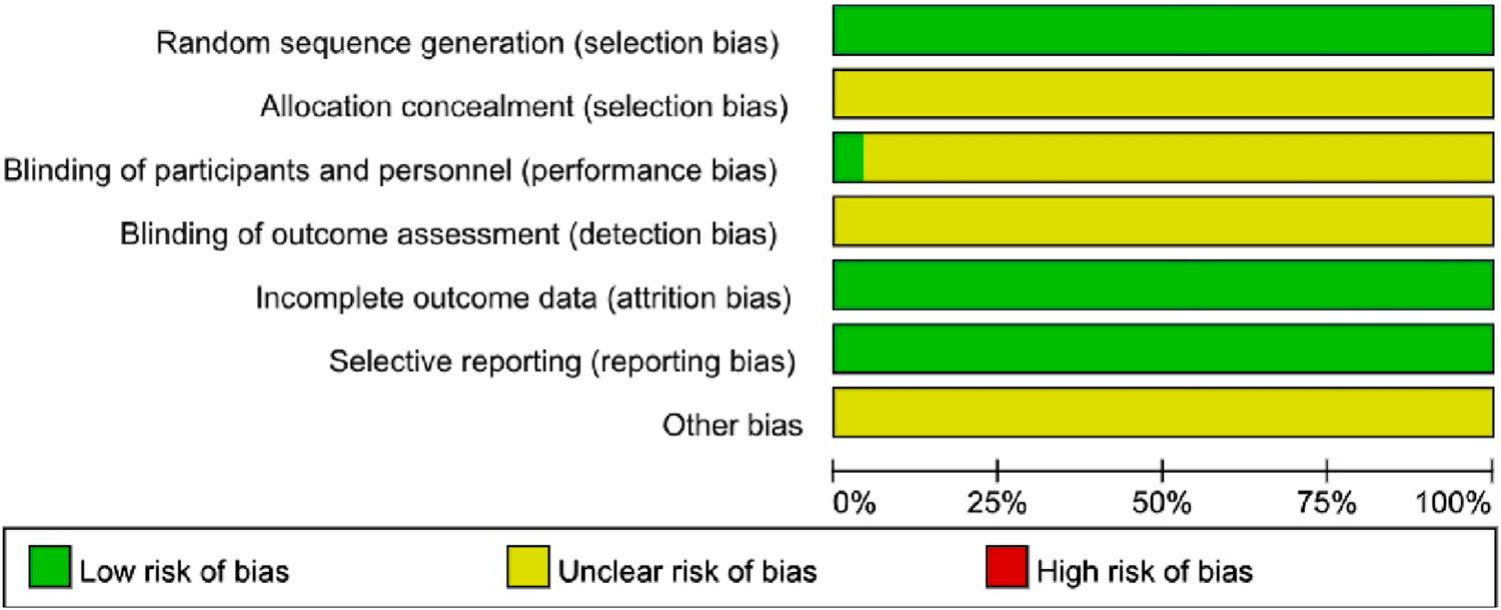
Risk of bias.

**FIGURE 3:**
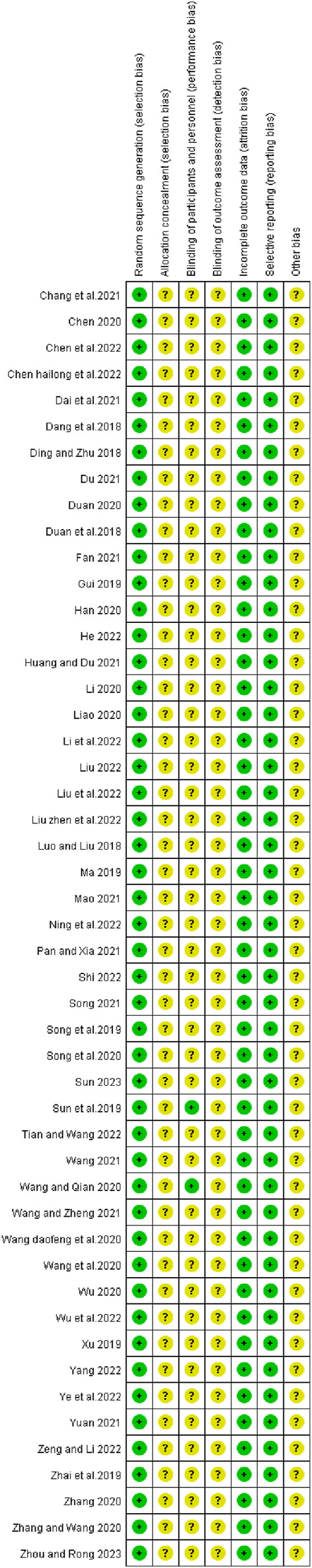
Risk of bias summary.

The assessment of the bias risk of these 49 RCTs was shown in Figure 2. All of these studies described specific random sequence generation. However, the detailed reporting of allocation, concealment, and blinding of outcome assessment had not been addressed in any studies. As for performance bias, it was mentioned in only two studies. The results of each study had been faithfully reported, so we consider all studies to be free of reporting bias (Figures 2, 3).

### Tumor response

**FIGURE 4:**
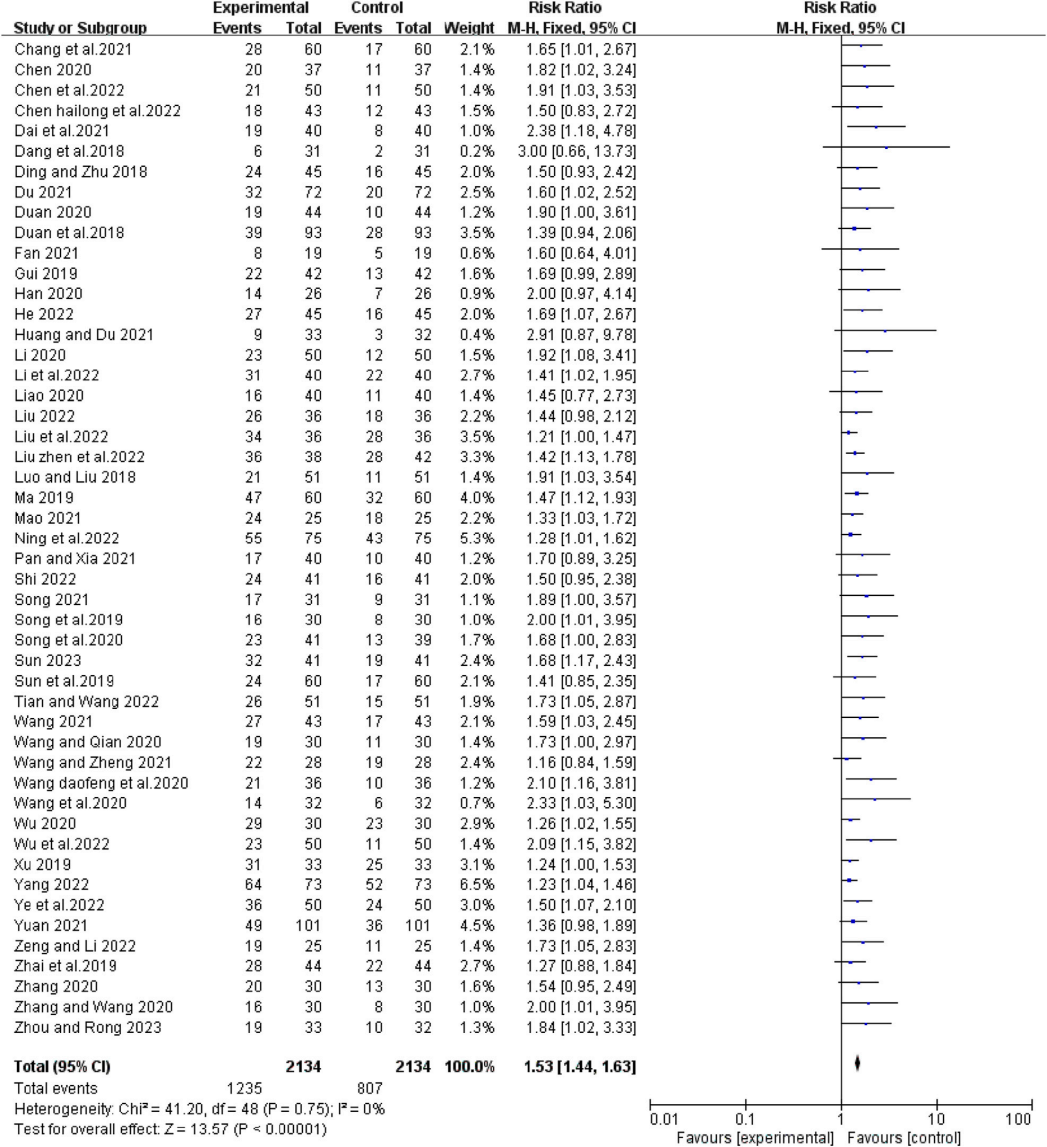
The pooled effects of bevacizumab combined with platinum-containing chemotherapy on objective response rate.

**FIGURE 5:**
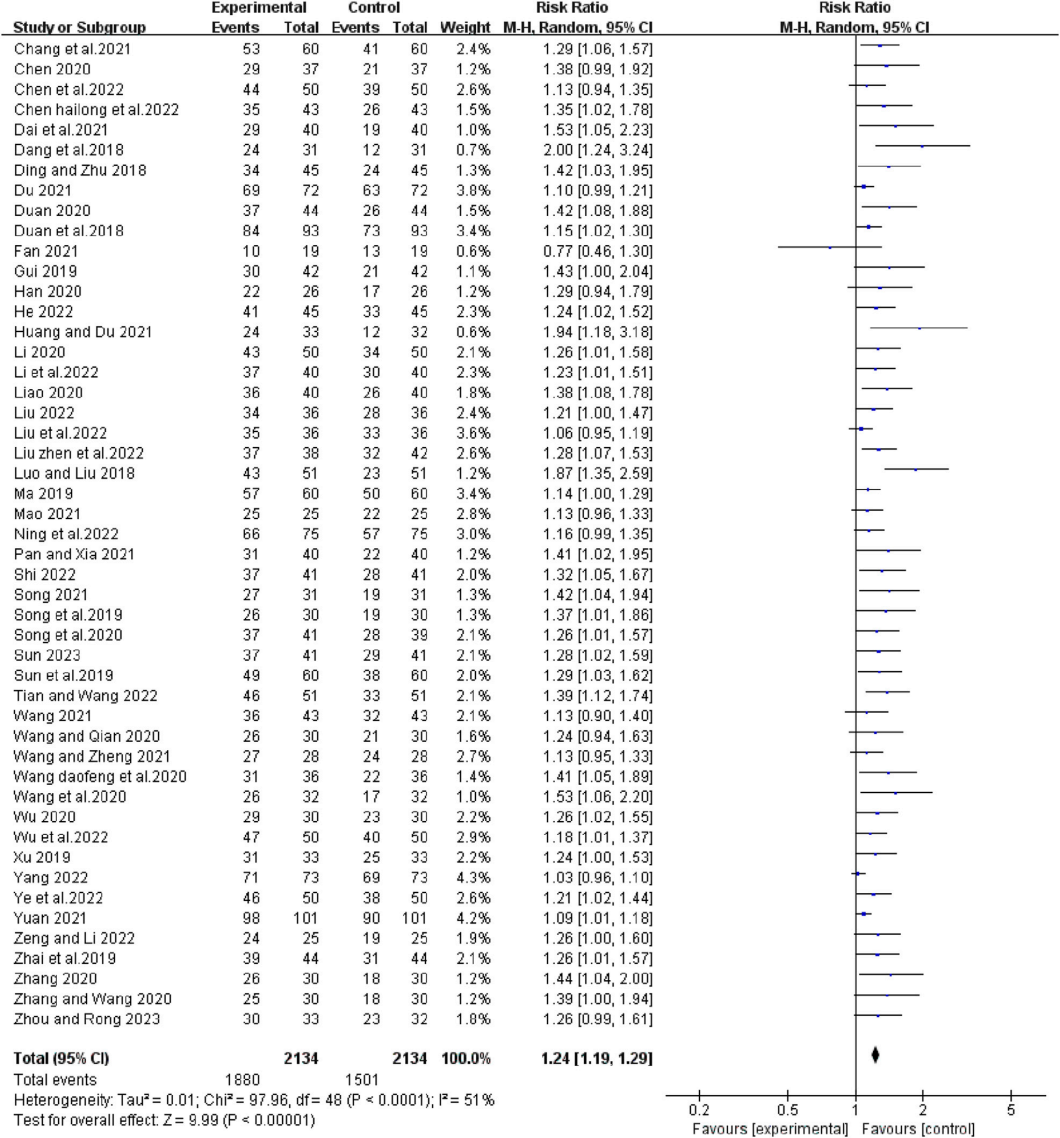
The pooled effects of bevacizumab combined with platinum-containing chemotherapy on disease control rate.

Forty-nine studies reported short-term efficacy of bevacizumab in combination with platinum-containing chemotherapy for advanced NSCLC. As there was no heterogeneity (χ^2^ = 41.20, *p* = 0. 75, I^2^ = 0%; Z = 13.57, *p* < 0.00001), we chose a fixed effects model for the analysis. As shown in Figure 4, the objective response rate (ORR) was higher in the experimental group than in the control group (RR [95% CI], 1.53 [1.44, 1.63], *p* < 0.00001). For the disease control rate (DCR), the test of heterogeneity (χ^2^ = 97.96, *p* < 0.0001, I^2^ = 51%; Z = 9.99, *p* < 0.00001) suggested that there was significant heterogeneity, so the random models were employed to combine effect sizes. Compared with platinum-based chemotherapy alone, the addition of bevacizumab had a better effect in terms of DCR (RR [95% CI], 1.24 [1.19, 1.29], *p* < 0.0001) (Figure 5). We then performed regression and subgroup analyses, which showed no significant differences in evaluation criteria, chemotherapy agents, and first treatment (Supplementary Figures S1, S2).

### 1, 2, and 3-year survival rate

**FIGURE 6:**
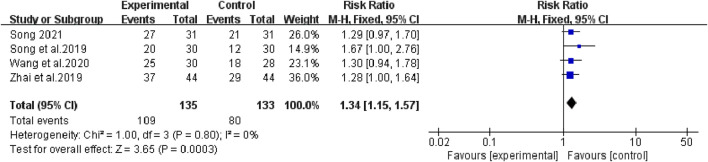
The pooled effects of bevacizumab combined with platinum-containing chemotherapy on 1-year survival rate.

**FIGURE 7:**
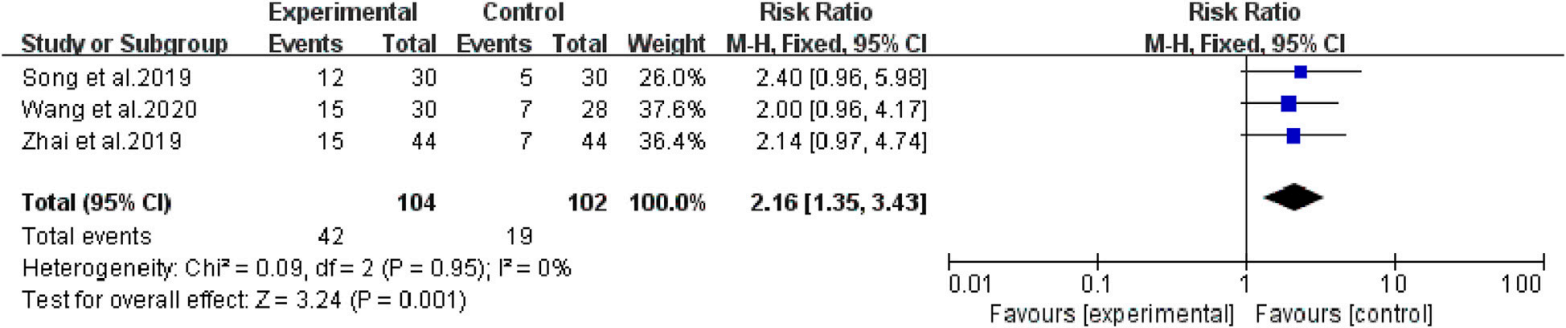
The pooled effects of bevacizumab combined with platinum-containing chemotherapy on 2-year survival rate.

**FIGURE 8:**
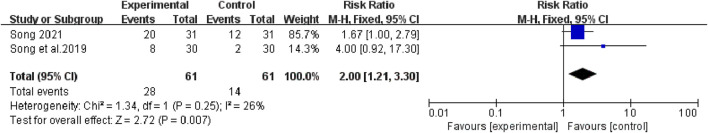
The pooled effects of bevacizumab combined with platinum-containing chemotherapy on 3-year survival rate.

The heterogeneity test suggested that there was no statistical heterogeneity among the studies, so the fixed effects model was selected. Four articles reported 1-year survival rate (χ^2^ = 1.00, *p* = 0.80, I^2^ = 0%; Z = 3.65, *p* = 0.0003), which showed that bevacizumab plus platinum-based chemotherapy was superior to chemotherapy alone (RR [95% CI], 1.34 [1.15, 1.57], *p* = 0.0003) (Figure 6). Three studies reported 2-year survival (χ^2^ = 0.09, *p* = 0.95, I^2^ = 0%; Z = 3.24, *p* = 0.001), which was superior with bevacizumab plus platinum-based chemotherapy (RR [95% CI], 2.16 [1.35, 3.43], *p* = 0.001) (Figure 7). Two studies reported 3-year survival (χ^2^ = 1.34, *p* = 0.25, I^2^ = 26%; Z = 2.72, *p* = 0.007), which was also significantly higher in the combination group (RR [95% CI], 2.00 [1.21, 3.30], *p* = 0.007) (Figure 8).

### VEGF levels

**FIGURE 9:**
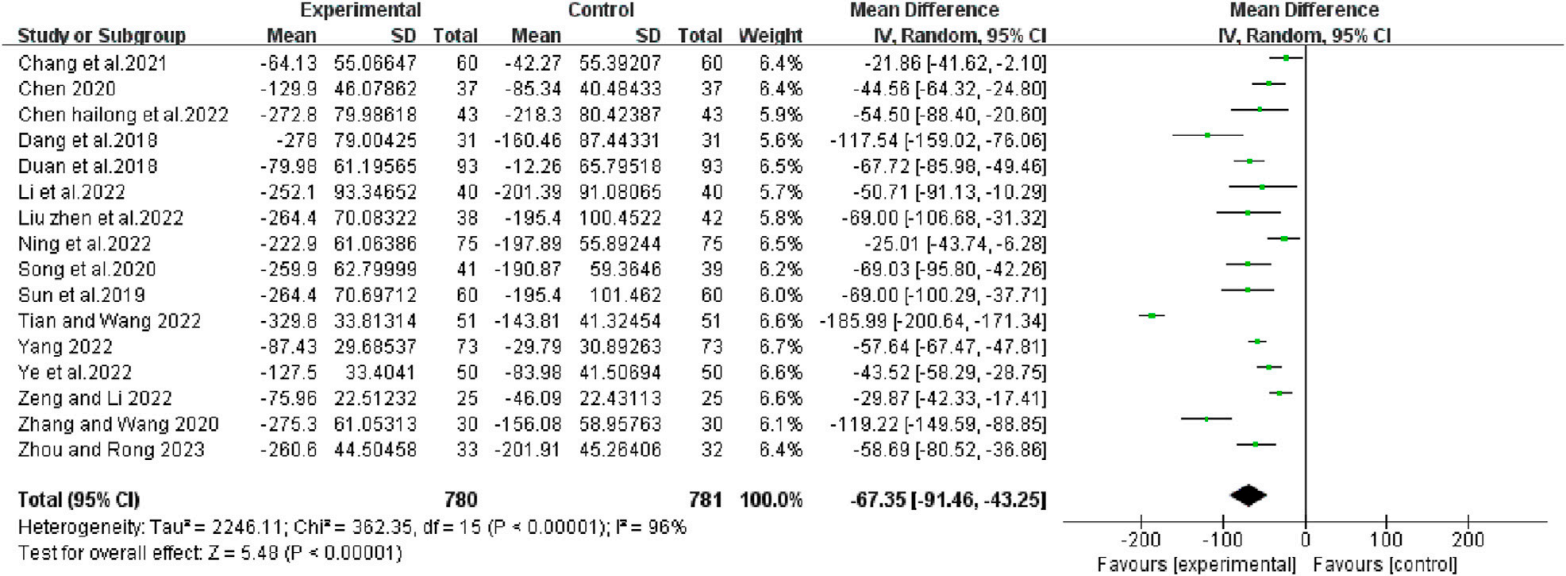
The pooled effects of bevacizumab combined with platinum-containing chemotherapy on VEGF leave.

Sixteen articles reported changes in serum VEGF levels before and after chemotherapy. The results of heterogeneity test showed that there was statistical heterogeneity among the studies (χ^2^ = 362.35, *p* < 0.00001, I^2^ = 96%; Z = 5.48, *p* < 0.00001), so a random effects model was chosen to combine effect sizes. The results of the analysis showed that the combination treatment group reduced VEGF levels better than the control group (RR [95% CI], −67.35 [−91.46, −43.25], *p* < 0.00001) (Figure 9).

### Side effects

The adverse reactions of chemotherapy involved in this study mainly included gastrointestinal reactions, bone marrow suppression, liver and kidney injury, and hematological toxicity. The results of Meta-analysis showed that there was no significant difference in the incidence of gastrointestinal reactions (RR [95% CI], 0.96 [0.88, 1.05], *p* = 0.38), myelosuppression (RR [95% CI], 0.95 [0.84, 1.09],*p* = 0.48), liver and kidney dysfunction (RR [95% CI], 0.92 [0.70, 1.20], *p* = 0.52) and hematologic toxicity (thrombocytopenia: RR [95% CI], 0.88 [0.72, 1.07], *p* = 0.20; leukopenia: RR [95% CI], 0.95 [0.81, 1.11], *p* = 0.49; hemoglobin reduction: RR [95% CI], 0.89 [0.71, 1.12], *p* = 0.31) between the experimental group and the control group (Supplementary Figures S3–S8).

### Publication bias and sensitivity analysis

**FIGURE 10:**
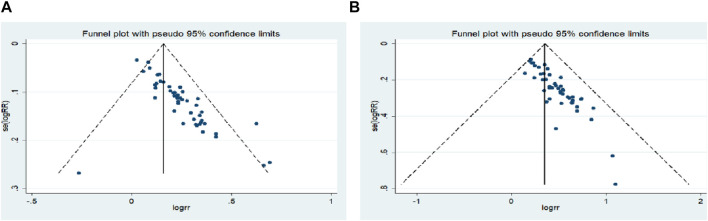
Begg`s regression analyses for publication bias: **(A)** Objective response rate; **(B)** Disease control rate.

**FIGURE 11:**
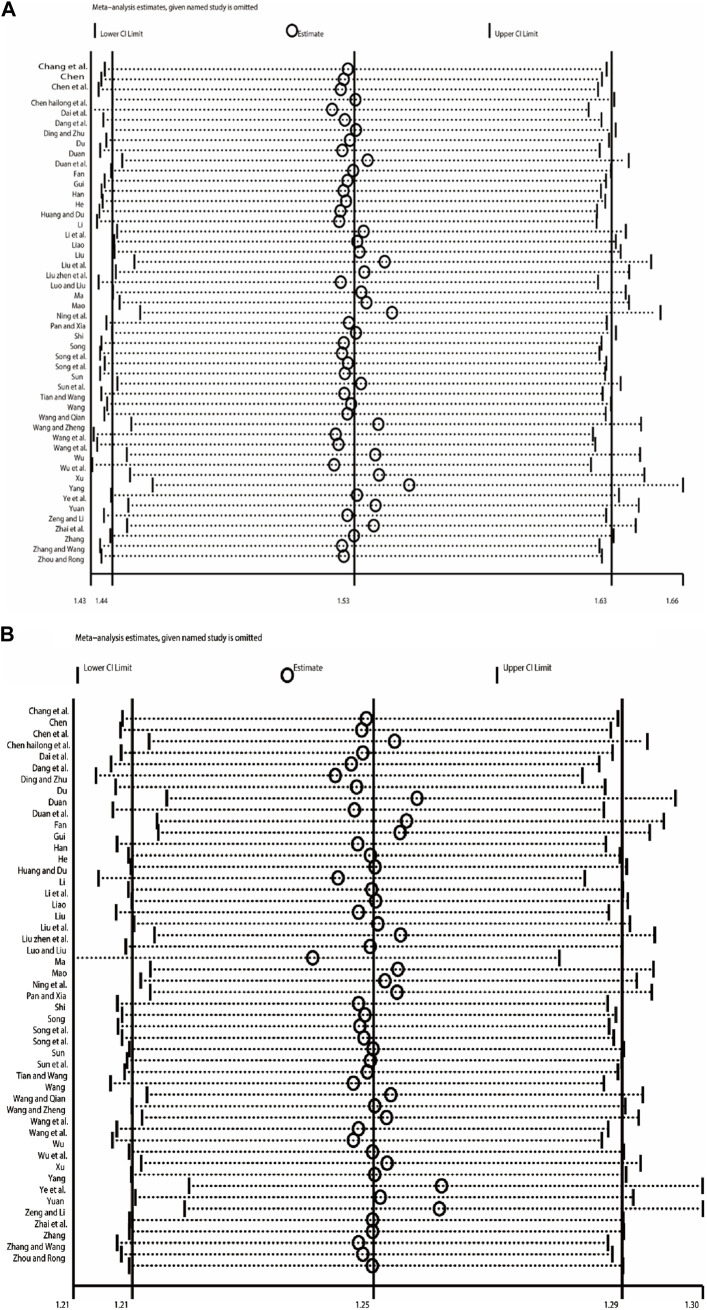
Sensitivity analysis plots: **(A)** Objective response rate; **(B)** Disease control rate.

Begg’s test was used to analyze the publication bias of ORR and DCR, and the results showed that publication bias may have a certain impact on the objective response rate and disease control rate (ORR: *p* = 0.000; DCR: *p* = 0.000) (Figure 10). Subsequently, sensitivity analysis was performed on outcome indicators such as objective response rate and disease control rate, and the results showed that the change of effect model had no significant effect on the combined results (Figure 11).

## Discussion

Despite major advances in molecular targeted therapy and immunotherapy in the past few years, platinum regimens remain the most active combination in clinical practice (Watanabe et al., 2017), Combination therapy has gained prominence in clinical practice. This systematic review and meta-analysis aim to assess the efficacy and safety of combining bevacizumab with platinum-containing chemotherapy in advanced NSCLC. The findings demonstrate superior objective response rate, disease control rate, 1/2/3-year survival rates, as well as a greater reduction in VEGF levels when compared to platinum-based chemotherapy alone. Furthermore, the combination therapy does not augment the adverse effects associated with chemotherapy alone while ensuring its safety is maintained.

Response Evaluation Criteria in Solid Tumors (RECIST) reflects the therapeutic effect of clinical tumors by detecting changes in tumor burden, including tumor shrinkage (objective response) and disease progression. ORR was evaluated as complete response (CR) and partial response (PR). DCR included CR or PR in all patients and stable disease (SD) in patients with progressive disease (PD) at the time of chemotherapy (Eisenhauer et al., 2009).

The 49 studies included in this meta-analysis reported ORR and DCR. The results of statistical analysis proved that bevacizumab combined with platinum-based chemotherapy had a significant advantage in improving ORR (RR 1.53) and DCR (RR 1.24) compared with platinum-based chemotherapy alone. The results of the ORR analysis are consistent with those of previous studies (Zhou et al., 2021). However, the significant improvement in DCR outcomes with the bevacizumab-containing therapy in our analysis is inconsistent with this finding. The study did not show significant outcomes with DCR, and the authors suggest that one of the studies may have influenced the results, acknowledging that the limited number of included RCTS limits the positive findings. Then subgroup analysis was performed according to the different chemotherapy drugs and whether they received anti-tumor treatment for the first time. Subgroup analyses showed no statistically significant differences between groups, indicating a significant benefit of bevacizumab-containing chemotherapy in improving DCR regardless of differences in the use of platinum-based chemotherapy agents or whether patients had received prior anticancer therapy.

For patients with advanced cancer, the annual survival rate is an important parameter in evaluating prognosis. In our analysis, the results of 1-year/2-year/3-year survival rates demonstrated a significant improvement in survival time for patients with advanced NSCLC when bevacizumab was combined with platinum compared to chemotherapy alone. However, due to limited literature included in of the evidence is relatively low, necessitating further data analysis to substantiate this conclusion in future studies.

Chemotherapy commonly used in clinical practice for NSCLC patients usually leads to serious adverse reactions, such as gastrointestinal reactions, bone marrow suppression, liver and kidney damage, etc. (Mangal et al., 2017) The results of our analysis show that the addition of bevacizumab to platinum-based chemotherapy does not increase the incidence of side effects of chemotherapy and confirm that bevacizumab has an acceptable safety profile, even when combined with different chemotherapy regimens. This conclusion is consistent with the results of the previous phase 4 study (Crinò et al., 2010).

Currently, extensive research on molecular targeted therapy for malignant tumors is being conducted in clinical practice, leading to of various targeted therapy drugs that have significantly improved the efficacy and control of malignant tumors. Studies have shown that neovascularization is closely related to the formation, development and prognosis of tumors. In the process of solid tumor growth, a variety of vascular growth factors are usually produced to promote the formation of new blood vessels. Among them, VEGF is considered to be the key factor inducing tumor angiogenesis. A number of studies have confirmed that VEGF is highly expressed in lung cancer, gastric cancer, intravascular sarcoma, gastrointestinal tumors and gynecological tumors (Lu et al., 2019). Blocking VEGF signaling can restore the vascular system to a more normal state, and improve the permeability of drugs in the tumor through the decrease of intercellular fluid pressure and the increase of tumor oxygenation, thereby enhancing the efficacy of chemotherapy (Jain, 2005). Bevacizumab can competitively bind to VEGF released by cancer tissues to impede angiogenesis within these tissues and inhibit tumor cell proliferation. Our findings suggest that bevacizumab combined with platinum-based chemotherapy can effectively reduce serum VEGF levels in patients with advanced NSCLC, which is consistent with the results of previous meta-analysis.

In conclusion, this meta-analysis showed that the combination of bevacizumab significantly improved the efficacy of chemotherapy and prolonged survival in patients with advanced non-small-cell lung cancer, without causing an increase in serious adverse effects.

### Limitations

All the studies included in this Meta-analysis were Chinese, which may lack sufficient representativeness and generalization across different countries. Further studies on different populations are needed to verify the generalizability of our findings. In addition, the description of distribution concealment and blinding was missing in most of the literature, resulting in a reduction in the quality of the included studies. The diversity of chemotherapeutic drugs also affected the results to a certain extent.

## Conclusion

Bevacizumab can enhance drug efficacy and improve the prognosis. In patients with advanced NSCLC, the efficacy of bevacizumab combined with platinum-based chemotherapy is better than that of single chemotherapy, and it does not increase the incidence of side effects of chemotherapy. These findings provide support for clinical treatment, suggesting that bevacizumab combination therapy in the treatment of advanced NSCLC has a significant benefit, with a modest safety profile. Further follow-up in the real world is needed to clarify its long-term benefits.

## Data Availability

The original contributions presented in the study are included in the article/Supplementary Material, further inquiries can be directed to the corresponding author.
