# Supplementary material for: The efficacy of bevacizumab combined with platinum-containing chemotherapy in the treatment of advanced non-small cell lung cancer in China: a systematic review and meta-analysis of randomized clinical trials
Source: Front Pharmacol. 2024 Jan 22;15:1293039. doi: 10.3389/fphar.2024.1293039 (PMC10839783; doi:10.3389/fphar.2024.1293039)
Supplement: Supplementary file 1 [file DataSheet1.docx]

Supplementary

. metareg logrr tool therapy first, wsse(_selogES) bsest(reml)

Meta-regression Number of obs = 49

REML estimate of between-study variance tau2 = .004504

% residual variation due to heterogeneity I-squared_res = 30.49%

Proportion of between-study variance explained Adj R-squared = -0.14%

Joint test for all covariates Model F(3,45) = 0.39

With Knapp-Hartung modification Prob > F = 0.7586

------------------------------------------------------------------------------

logrr | Coef. Std. Err. t P>|t| [95% Conf. Interval]

-------------+----------------------------------------------------------------

tool | -.007323 .0251413 -0.29 0.772 -.0579601 .0433142

therapy | -.0073114 .0197568 -0.37 0.713 -.0471036 .0324808

first | -.0311696 .0382383 -0.82 0.419 -.1081855 .0458463

_cons | .2789193 .075421 3.70 0.001 .1270137 .4308249

------------------------------------------------------------------------------

**FIGURE S1.** The regression result of bevacizumab -containing chemotherapy on DCR.

A


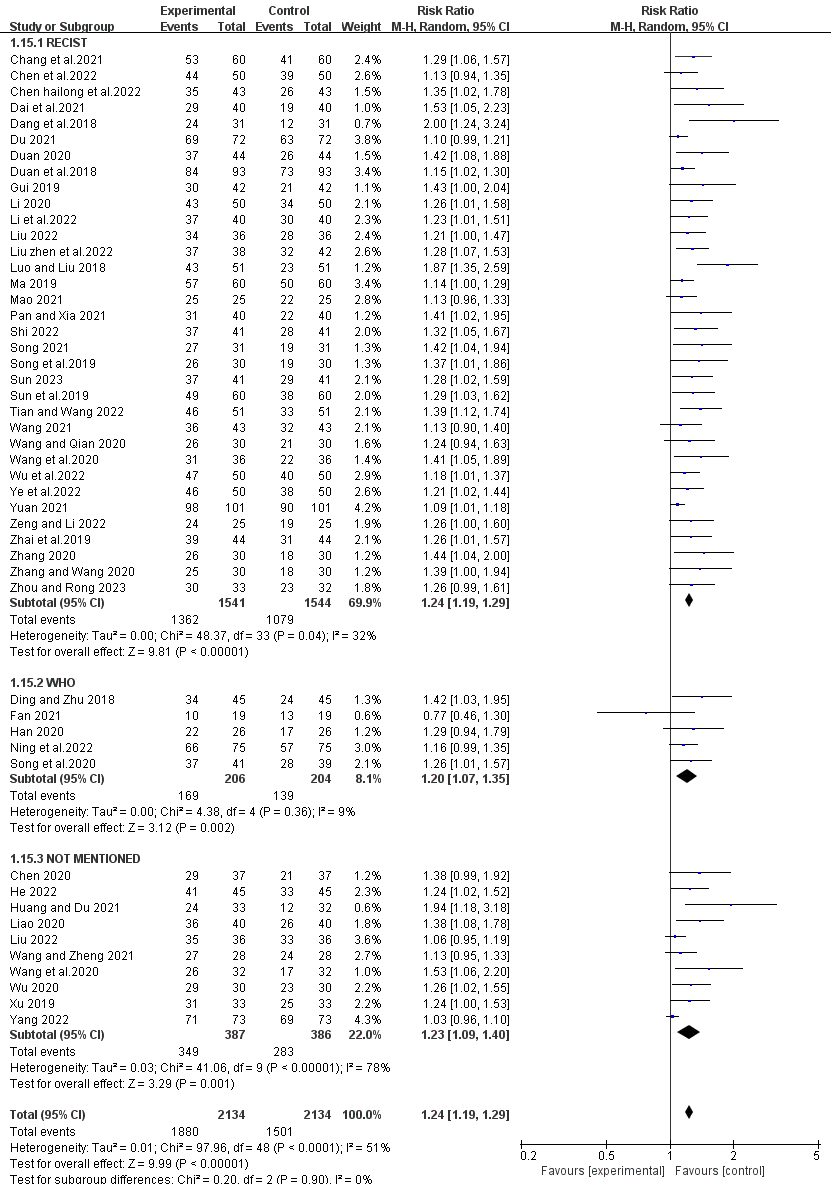


B


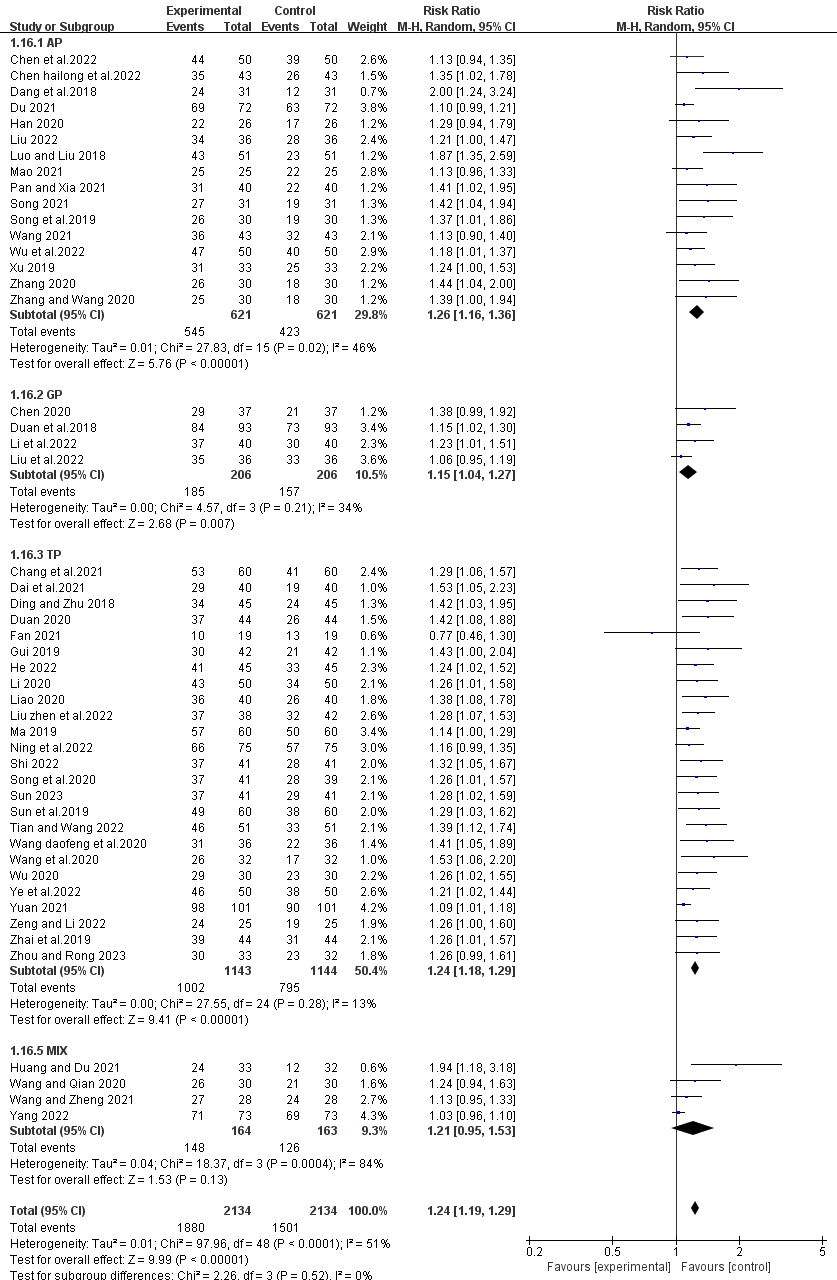


C


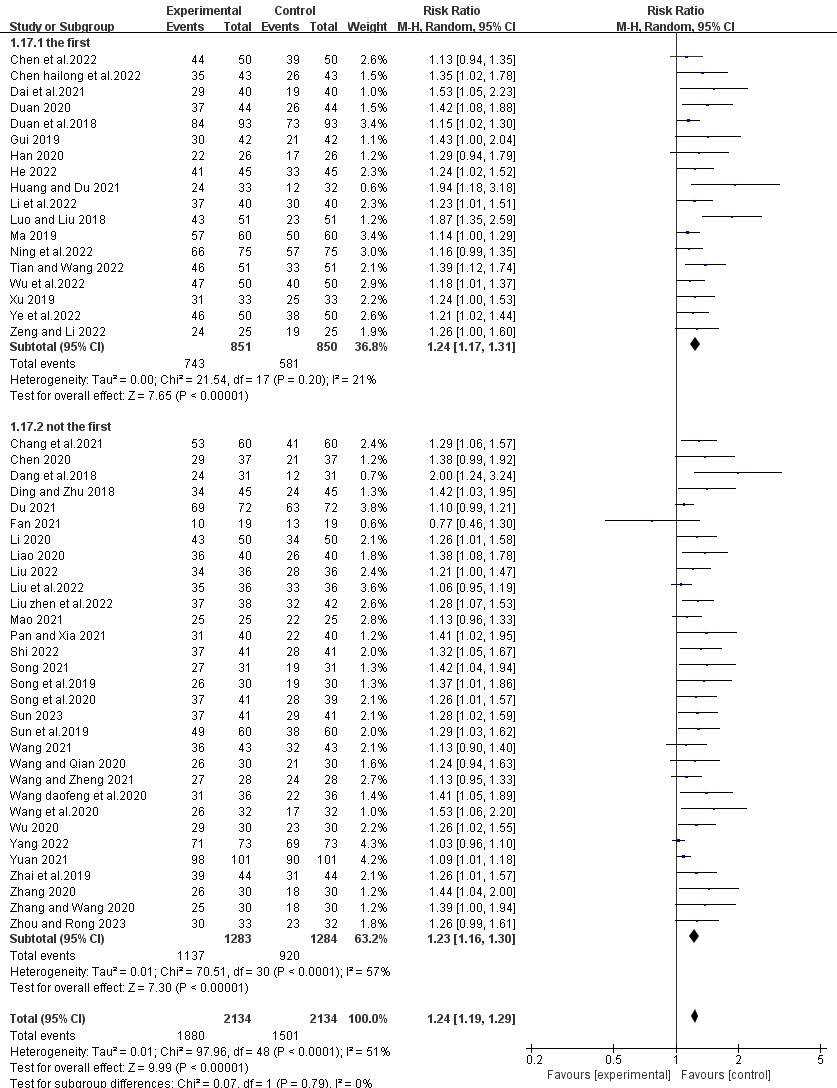


**FIGURE S2.** The subgroup analyses about (A) the evaluation criteria; (B) the drugs; (C) the first-treatment on DCR.


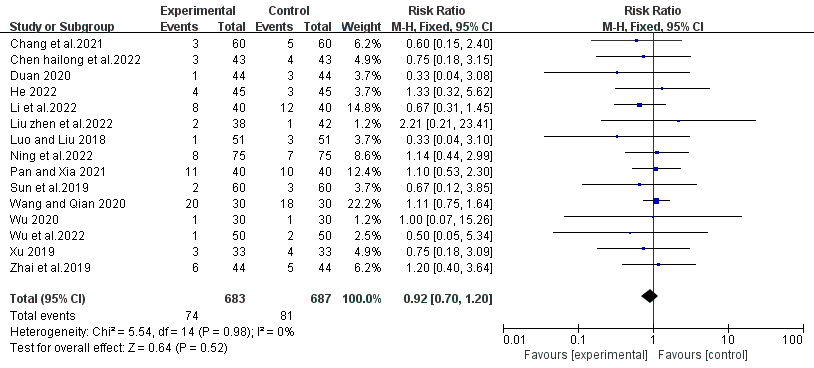


**FIGURE S3.** The pooled effects of bevacizumab -containing chemotherapy on liver and kidney abnormalities.


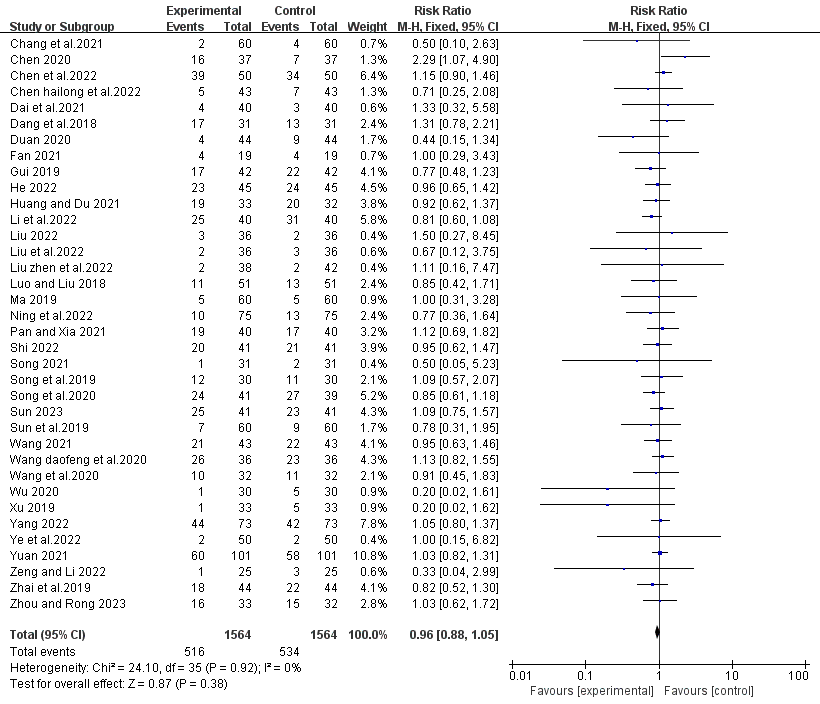


**FIGURE S4.** The pooled effects of bevacizumab -containing chemotherapy on gastrointestinal reactions.


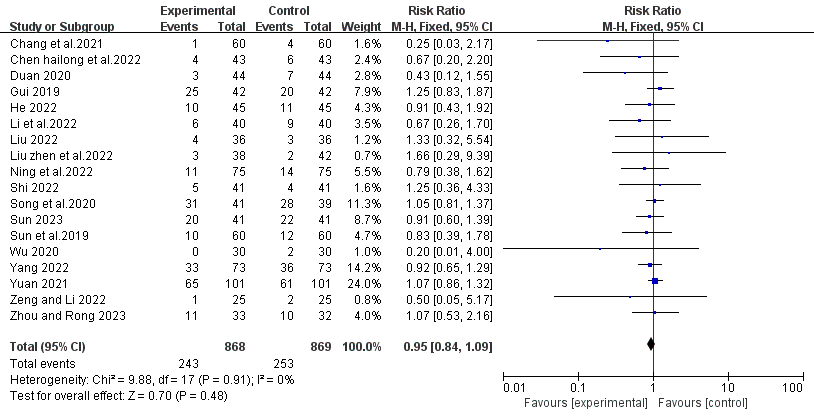


**FIGURE S5.** The pooled effects of bevacizumab -containing chemotherapy on bone marrow suppression.


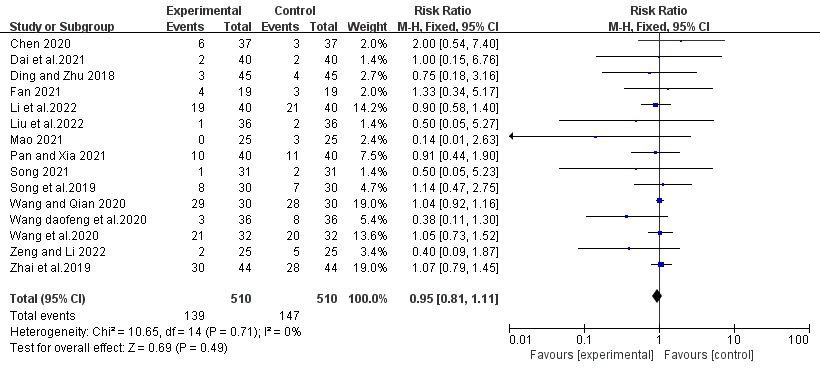


**FIGURE S6.** The pooled effects of bevacizumab -containing chemotherapy on leukopenia.


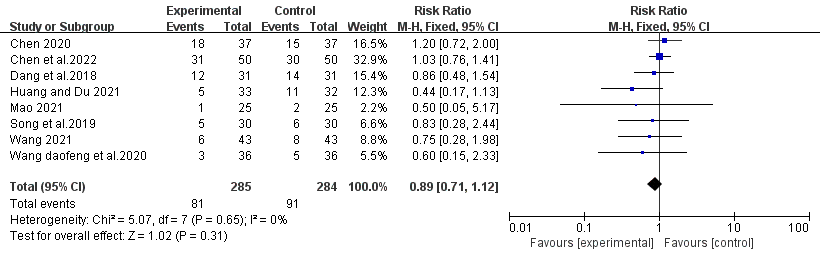


**FIGURE S7.** The pooled effects of bevacizumab -containing chemotherapy on reduced hemoglobin.


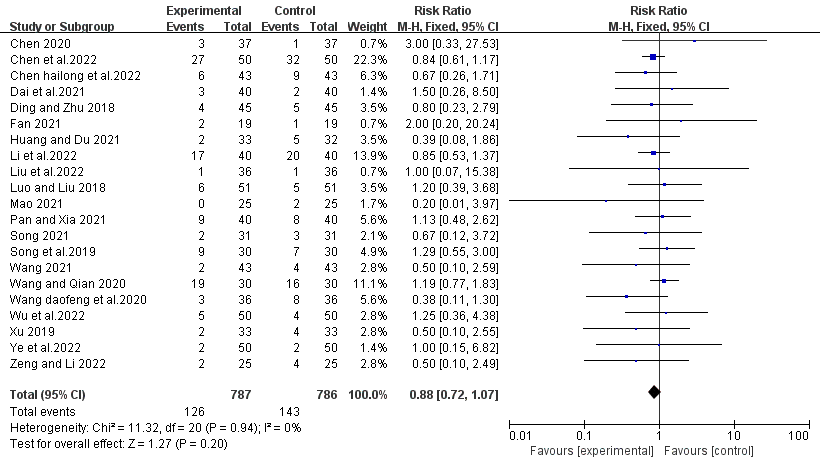


**FIGURE S8.** The pooled effects of bevacizumab -containing chemotherapy on thrombocytopenia.
